# Durvalumab after definitive chemoradiotherapy in locally advanced NSCLC: Data of the German EAP

**DOI:** 10.1016/j.dib.2020.106556

**Published:** 2020-12-05

**Authors:** Martin Faehling, Christian Schumann, Petros Christopoulos, Petra Hoffknecht, Jürgen Alt, Marlitt Horn, Stephan Eisenmann, Anke Schlenska-Lange, Philipp Schütt, Felix Steger, Wolfgang M. Brückl, Daniel C. Christoph

**Affiliations:** a Klinik für Kardiologie und Pneumologie, Klinikum Esslingen, 73730 Esslingen, Germany; b Klinik für Pneumologie, Thoraxonkologie, Schlaf- und Beatmungsmedizin, Klinikum Kempten, 87439 Kempten, Germany; c Thoraxklinik-Heidelberg, 69126 Heidelberg, Germany; d Klinik für Thoraxonkologie und Palliativstation, Franziskus-Hospital Harderberg, 49124 Georgsmarienhütte, Germany; e III. Medizinische Klinik und Poliklinik, Johannes Gutenberg-Universität, 55131 Mainz, Germany; f LungenClinic Grosshansdorf, Großhansdorf, Germany; g Universitätsklinik und Poliklinik für Innere Medizin I (Gastroenterologie & Pneumologie), Universitätsklinikum Halle (Saale), 06120 Halle, Germany; h Klinik für Onkologie und Hämatologie, Krankenhaus Barmherzige Brüder Regensburg, 93049 Regensburg, Germany; i Onkologische Gemeinschaftspraxis, Gütersloh, Germany; j Klinik und Poliklinik für Strahlentherapie, Universitätsklinikum Regensburg, 93053 Regensburg, Germany; k Klinik für Innere Medizin 3, Klinikum Nürnberg Nord, 90419 Nürnberg, Germany; l Klinik für Internistische Onkologie und Hämatologie mit integrierter Palliativmedizin, Evang. Kliniken Essen-Mitte, 45136 Essen, Germany

**Keywords:** NSCLC, PD-L1, Checkpoint inhibitor, Survival, Real world, Oligometastatic, Autoimmune

## Abstract

Following the PACIFIC trial, durvalumab has been approved by the European Medicines Agency (EMA) for consolidation of locally advanced PD-L1-positive NSCLC after chemoradiotherapy (CRT). Patients were treated with durvalumab in the EAP from 22.11.2017 to 15.10.2018 allowing analysis of its efficacy and safety.

211 patients were registered by 90 German centres. Data were collected retrospectively by questionnaire and queries. 56 centres reported data on 126 patients who actually received at least one cycle of durvalumab. In contrast to the PACIFIC-trial population, some patients with oligometastatic disease and a history of autoimmune disease are included in the EAP population. Information on PD-L1 status was obtained for 111 patients. Baseline data include age, gender, ECOG, stage (IASLC 8th ed.), and smoking history. Treatment data include mode of chemoradiotherapy, used chemotherapy agent, and duration of durvalumab therapy. Adverse evants were documented according to CTAEC 5.0. Data were analysed for progression-free survival (PFS), overall survival (OS), and adverse events (AE). The results were published in Lung Cancer [1].

## Specifications Table

**Table:** 

Subject	Oncology
Specific subject area	Thoracic oncology, NSCLC locally advanced or oligometastatic disease
Type of data	1 text document (survey)
	1 data Table
	1 figure
	2 tables
How data were acquired	Data were acquired by survey.
	Analysis was performed using Excel and Graph pad prism.
Data format	Survey: docx
	Raw data: Excel
	Figure: (embedded)
	Tables: (embedded)
Parameters for data collection	Date of diagnosis, last contact, vital status, age, weight, size, smoking history, ECOG at start of durvalumab, stage of NSCLC, histology, PD-L1 (TPS), history of autoimmune disease, type of radiochemotherapy, chemotherapy used, dates of durvalumab treatment, recurrence: site, date, adverse events.
Description of data collection	Survey and queries.
Data source location	Institution: Klinikum Esslingen
	City/Town/Region: Esslingen
	Country: Germany
Data accessibility	With the article
	Instructions for accessing these data: open access.
Related research article	Authors’ names: Martin Faehling, Christian Schumann, Petros Christopoulos, Petra Hoffknecht, Jürgen Alt, Marlitt Horn, Stephan Eisenmann, Anke Schlenska-Lange, Philipp Schütt, Felix Steger, Wolfgang M. Brückl, Daniel C. Christoph.
	Title: Durvalumab after definitive chemoradiotherapy in locally advanced unresectable Non-small cell lung cancer (NSCLC): Real-world data on survival and safety from the German expanded-access program (EAP)
	Journal: Lung Cancer. 2020;150:114–122.
	https://doi.org/10.1016/j.lungcan.2020.10.006.

## Value of the Data

• The data describes the largest multicentric national cohort with detailed clinical characteristics and longest follow-up so far published. The national cohort comprises more patients than the German subgroup of the PACIFIC-trial.
• The data is of interest for thoracic oncologists studying locally advanced or oligometastatic NSCLC
• The data might be used for pooled analysis with data from other sources on rare subgroups (e. g. oligometastatic NSCLC) or subgroups not well represented in prospective trials (e. g. patients with autoimmune disease). for cross-country comparisons of treatment standards and outcome with data sets from other countries.
• These data provide real world information on the use of durvalumab in Europe.
• These data provide real world information on the use of durvalumab in subgroups not included in clinical trials (oligometastatic stage-IVA patients, patients with stable autoimmune diseases).
• The pooled analysis of rare subgroups could provide the basis for improved treatment of subgroups for which prospective trial data are lacking.

## 1 Data Description

The data describe overall survival of subgroups of patients treated with durvalumab consolidation after definitive chemoradiotherapy by age, gender, performance status (ECOG 0,1,2), histology (adenocarcinoma, squamous-cell carcinoma, other), mode of chemoradiotherapy (with or without induction chemotherapy), or chemotherapy used (cisplatin or carboplatin). Sites of recurrence are given in Data Table 1. The survival data are summarized as Kaplan-Meier curves (Data Fig. 1), baseline characteristics of each subgroup are provided in the respective tables (Data Table 2A, Data Table 2B, Data Table 2C, Data Table 2D).

**Data Fig. 1 (pdf):**
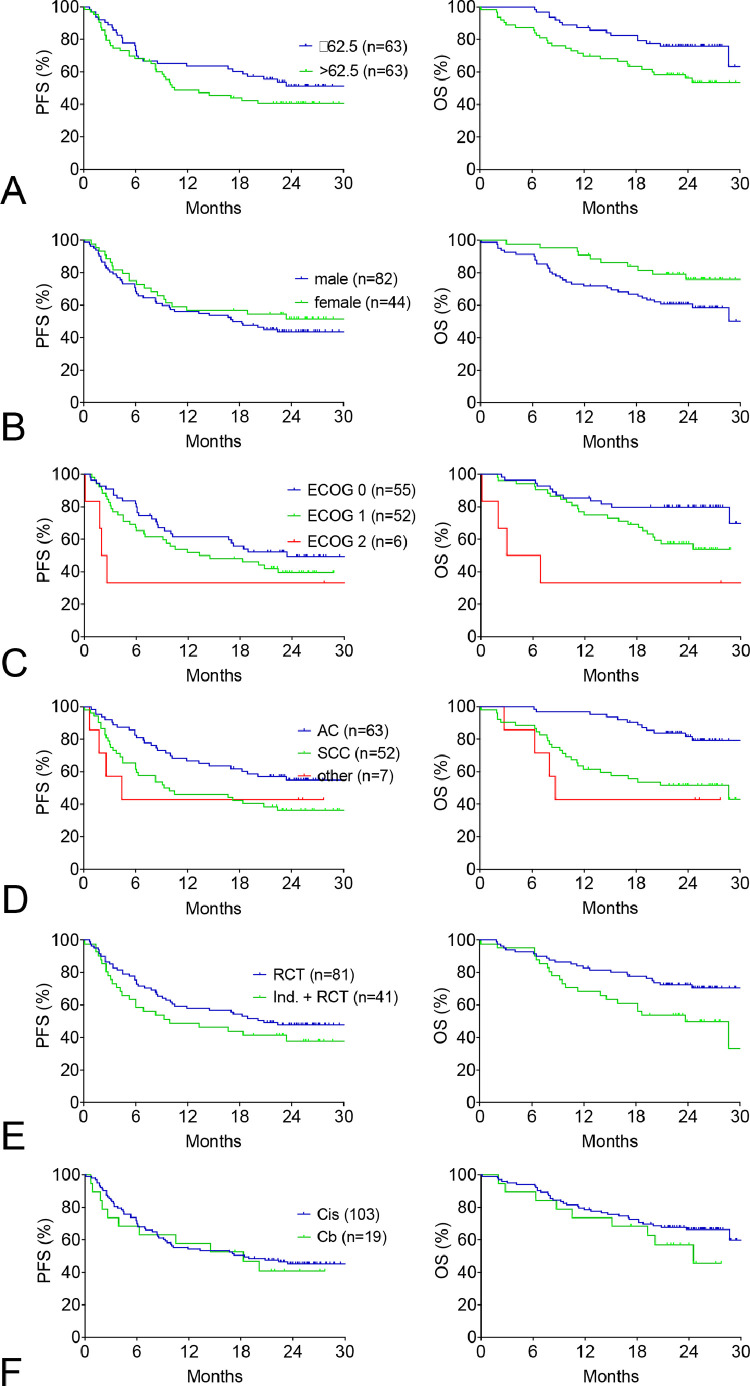
Survival of subgroups of the EAP population from start of durvalumab.

**Data Table 1:** Sites of recurrence.

n	126
Patients with recurrence	59
*Number of recurrence sites*	
Only one site	32
2 sites	17
3–4 sites	8
*Sites of recurrence*	
*Intrathoracic recurrence only*	*32*
Local recurrence (within radiation field)	25
Local recurrence only	13
Lung	23
Lung only	11
Pleura	9
Pleura only	1
*Extrathoracic recurrence*	*27*
Brain	8
Brain only	3
Bone	10
Bone only	0
Liver	5
Liver only	2
Adrenals	6
Adrenal only	2
Extra thoracic lymph nodes	7
Lymph nodes only	0
Other^1^	4
Other only^2^	1

1 3: soft tissue, 1: pancreas and spleen.

2 2: soft tissue.

**Data Table 2A:** Age.

	Age	
	≤62.5 years	>62.5 years
**n**	63	63
**Age (mean, range)**	55.5 (33.5 – 62.5)	69.3 (62.7– 81.6)
***Gender***		
Male	39 (62%)	43 (68%)
Female	24 (38%)	20 (32%)
***Stage (UICC 8)***		
IIIA	8 (13%)	25 (40%)
IIIB	37 (59%)	18 (29%)
IIIC	15 (24%)	16 (25%)
IVA	1 (2%)	4 (6%)
IVB	2 (3%)	0
***Performance status***	*NA 4 (6%)*	*NA 9 (14%)*
ECOG 0	35 (59%)	20 (37%)
ECOG 1	23 (39%)	29 (54%)
ECOG 2	1 (2%)	5 (9%)
***Histology***	*NA 2 (3%)*	*NA 2 (3%)*
Adenocarcinoma	35 (57%)	28 (46%)
Squamous cell carcinoma	22 (36%)	30 (49%)
Adenosquamous carcinoma	0	1 (2%)
LCNEC	2 (3%)	1 (2%)
NOS	2 (3%)	1 (2%)
***PD-L1 (%)***	*NA 5 (8%)*	*NA 10 (16%)*
0	19 (33%)	13 (25%)
1 - 49	18 (31%)	24 (45%)
50 - 100	21 (36%)	16 (30%)
***Deceased***	16 (25%)	28 (44%)
Death related to NSCLC	14 (22%)	18 (29%)
Death unrelated to NSCLC	2 (3%)	10 (16%)

**Data Table 2B:** Gender.

	Gender	
	male	female
**n**	82	44
**Age (mean, range)**	63.3 (44.8 – 81.6)	60.7 (33.5 – 78.9)
***Gender***		
Male	82	0
Female	0	44
***Performance status***	NA 8	NA 5
ECOG 0	34 (46%)	21 (54%)
ECOG 1	37 (50%)	15 (39%)
ECOG 2	3 (4%)	3 (8%)
***Stage (UICC 8)***		
IIIA	21 (26%)	12 (27%)
IIIB	35 (43%)	20 (46%)
IIIC	23 (28%)	8 (18%)
IVA	3 (4%)	2 (5%)
IVB	0	2 (5%)
***Histology***	NA 2 (2%)	NA 2 *(5%)*
Adenocarcinoma	33 (41%)	30 (71%)
Squamous cell carcinoma	41 (51%)	11 (26%)
Adenosquamous carcinoma	1 (1%)	0
LCNEC	2 (3%)	1 (2%)
NOS	3 (4%)	0
***PD-L1 (%)***	NA 12 *(15%)*	NA 3 *(7%)*
0	21 (30%)	11 (28%)
1 - 49	27 (39%)	15 (37%)
50 - 100	22 (31%)	15 (37%)
***Deceased***	34 (41%)	10 (23%)
Death related to NSCLC	22 (27%)	10 (23%)
Death unrelated to NSCLC	12 (15%)	0

**Data Table 2C:** Performance status.

*NA 13 (10%)*	Performance status		
	ECOG 0	ECOG 1	ECOG 2
**n**	55	52	6
**Age (mean, range)**	60.8 (33.5 – 77.7)	63.9 (47.9 – 81.6)	68.4 (50.8 – 78.9)
***Gender***			
Male	34 (62%)	37 (71%)	3 (50%)
Female	21 (38%)	15 (29%)	3 (50%)
***Stage (UICC 8)***			
IIIA	12 (22%)	15 (29%)	2 (33%)
IIIB	24 (44%)	23 (44%)	2 (33%)
IIIC	16 (29%)	10 (19%)	2 (33%)
IVA	1 (2%)	4 (8%)	0
IVB	2 (4%)	0	0
***Histology***	*NA 2 (4%)*	*NA 1 (2%)*	*NA 1 (17%)*
Adenocarcinoma	33 (62%)	24 (47%)	0
Squamous cell carcinoma	17 (32%)	24 (47%)	4 (80%)
Adenosquamous carcinoma	1 (2%)	0	0
LCNEC	1 (2%)	1 (2%)	1 (20%)
NOS	1 (2%)	2 (4%)	0
***PD-L1 (%)***	*NA 2 (4%)*	*NA 11 (21%)*	*NA 1 (17%)*
0	14 (26%)	11 (27%)	1(20%)
1 - 49	19 (36%)	18 (44%)	2 (40%)
50 - 100	20 (38%)	12 (29%)	2 (40%)
***Deceased***	12 (22%)	23 (44%)	4 (67%)
Death related to NSCLC	9 (16%)	16 (31%)	2 (33%)
Death unrelated to NSCLC	3 (5%)	7 (13%)	2 (33%)

**Data Table 2D:** Histology.

	Histology NA 5		
	Adenocarcinoma	Squamous cell carcinoma	other
**n**	63	52	7
***Age (mean, range)***	60.8 (33.5 – 77.7)	64.5 (47.9 – 81.6)	59.9 (44.8 – 73.4)
***Gender***			
Male	33 (52%)	41 (79%)	6 (86%)
Female	30 (48%)	11 (21%)	1 (14%)
***Stage (UICC 8)***			
IIIA	20 (32%)	11 (21%)	2 (29%)
IIIB	29 (46%)	23 (44%)	1 (14%)
IIIC	10 (16%)	16 (31%)	3 (43%)
IVA	2 (3%)	2 (4%)	1 (14%)
IVB	2 (3%)	0	0
***Performance status***	*NA 6 (10%)*	*NA 7 (13%)*	
ECOG 0	33 (58%)	17 (38%)	3 (43%)
ECOG 1	24 (42%)	24 (53%)	3 (43%)
ECOG 2	0	4 (9%)	1 (14%)
***Histology***			
Adenocarcinoma	63	0	0
Squamous cell carcinoma	0	52	0
Adenosquamous carcinoma	0	0	1
LCNEC	0	0	3
NOS	0	0	3
***PD-L1 (%)***	*NA 7 (11%)*	*NA 5 (10%)*	*NA 3 (43%)*
0	12 (21%)	17 (36%)	2 (50%)
1 - 49	18 (32%)	20 (43%)	1 (25%)
50 - 100	26 (46%)	10 (21%)	1 (25%)
***Deceased***	12 (19%)	26 (50%)	4 (57%)
Death related to NSCLC	10 (16%)	16 (31%)	4 (57%)
Death unrelated to NSCLC	2 (3%)	10 (19%)	0

**Data Table 2E:** Mode of RCT and prior chemotherapy of patients treated with simultaneous CRT with or without induction chemotherapy. Four patients (3.2%) had received chemotherapy and radiotherapy sequentially and were not analysed separately.

	RT mode, excluding sequential RCT *n* = 4)		
	RCT only	Induction + RCT	
**n**	81	41	
**Age (mean, range)**	62.3 (33.5 – 81.6)	62.2 (46.6 – 77.1)	
***Gender***			
Male	50 (62%)	29 (71%)	
Female	31 (38%)	12 (29%)	
***Performance status***	NA 7	NA 6	
ECOG 0	38 (51%)	16 (46%)	
ECOG 1	34 (46%)	16 (46%)	
ECOG 2	2 (3%)	3 (9%)	
***Smoking status***	NA 4		
Never-smoker	4 (5%)	1 (2%)	
Ever smoker	73 (95%)	40 (98%)	
Pack years (mean, range)	41 (7.5 – 120)	43 (8 – 80)	
***Histology***	NA 3 (4%)	*NA 1 (2%)*	
Adenocarcinoma	46 (59%)	15 (38%)	
Squamous cell carcinoma	28 (36%)	22 (55%)	
Adenosquamous carcinoma	1 (1%)	0	
LCNEC	1 (1%)	2 (5%)	
NOS	2 (3%)	1 (2%)	
***Stage (UICC 8)***			
IIIA	21 (26%)	9 (22%)	
IIIB	42 (52%)	13 (32%)	
IIIC	16 (20%)	14 (34%)	
IVA	1 (1%)	4 (10%)	
IVB	1 (1%)	1 (2%)	
***PD-L1 (%)***	*NA 8 (10%)*	*NA 5 (12%)*	
0	20 (27%)	12 (33%)	
1 - 49	25 (34%)	15 (42%)	
50 - 100	28 (38%)	9 (25%)	
***Chemotherapy***	RCT only	Ind. CT	RCT after ind. CT
**Platinum:**		*NA 2 (5%)*	
Cisplatin	66 (81%)	33 (85%)	37 (90%)
Carboplatin	15 (19%)	6 (15%)	4 (10%)
**Combination agent:**			
Vinorebine	62 (77%)	9 (23%)	30 (73%)
Paclitaxel	7 (9%)	18 (46%)	5 (12%)
nab-Paclitaxel	1 (1%)	3 (8%)	0
Pemetrexed	4 (5%)	3 (8%)	3 (7%)
Docetaxel	0	0	0
Gemcitabine	0	4 (10%)	0
Etopside	3 (4%)	1 (3%)	1 (2%)
None (platin only)	4 (5%)	1 (3%)	2 (5%)
**Deceased**	23 (28%)	21 (51%)	
Death related to NSCLC	17 (21%)	15 (37%)	
Death unrelated to NSCLC	6 (7%)	6 (15%)	

The Kaplan-Meier plots on the left side show PFS, those on the right show OS. For clinical characteristics of the subgroups, compare Data Table 2. For numerical values of HRs, CIs, and significance levels, compare Table 4 of the *Lung Cancer* manuscript [1].

A. Age.
B. Gender.
C. Performance status.
D. Histology subgroups.
E. Mode of RCT and prior chemotherapy of patients treated with simultaneous CRT with or without induction chemotherapy. Four patients (3.2%) had received chemotherapy and radiotherapy sequentially and were not analysed separately.
F. Patients treated with cisplatin or carboplatin as part of the simultaneous CRT.

Data Table 2

Baseline characteristics and number of deaths of subgroups. There were no relevant differences among the subgroups with respect to smoking history or proportion of patients with a history of autoimmune diseases. All subgroups had a proportion of smokers of 94% - 100% with mean PY of 37 – 52, and a proportion of patients with an autoimmune disease of 0 - 14%. For better clarity, these parameters were not included in the subgroup tables.

A. Age.

B. Gender.

C. Performance status.

D. Histology.

E. Mode of CRT and prior chemotherapy of patients treated with simultaneous CRT with or without induction chemotherapy. Four patients (3.2%) had received chemotherapy and radiotherapy sequentially and were not analysed separately.

F. Patients treated with cisplatin or carboplatin as part of the simultaneous CRT.

Raw data file (MS excel) *File:* Faehling PACIFIC EAP Germany.xlsx

Excel file with raw data which were used for the analysis published in lung cancer:

Questionaireused to collect the data: *File:* Faehling CRF EAP Durvalumab final.docx

## 2 Experimental Design, Materials and Methods

German centres who registered patients for treatment with durvalumab in the Early Access Programme (EAP) were asked to report pseudonymized data on their patients using the questionnaire. The data were clarified using queries by mail. The data were transferred into the excel data file. The data were analysed using GraphPad Prism8. Kaplan-Meier plots were generated using GraphPad Prism8. HRs and 95% confidence intervals (CIs) were calculated using the log-rank (Mantel-Cox) test-algorithm of GraphPad Prism8.

**Data Table 2F:** Patients treated with cisplatin or carboplatin as part of the simultaneous CRT.

	Platinum (excl. sequential RCT, *n* = 4)	
	Cisplatin	Carboplatin
**n**	103	19
**Age (mean, range)**	61.2 (33.5 – 78.6)	68.0 (51.1 – 81.6)
***Gender***		
Male	68 (66%)	11 (58%)
Female	35 (34%)	8 (42%)
***Stage (UICC 8)***		
IIIA	24 (23%)	6 (32%)
IIIB	46 (45%)	9 (47%)
IIIC	26 (25%)	4 (21%)
IVA	5 (5%)	0
IVB	2 (2%)	0
***Performance status***	*NA 9 (9%)*	*NA 4 (21%)*
ECOG 0	51 (54%)	3 (20%)
ECOG 1	39 (42%)	11 (73%)
ECOG 2	4 (4%)	1 (7%)
***Histology***	*NA 2 (2%)*	*NA 2 (11%)*
Adenocarcinoma	54 (53%)	7 (41%)
Squamous cell carcinoma	40 (40%)	10 (59%)
Adenosquamous carcinoma	1 (1%)	0
LCNEC	3 (3%)	0
NOS	3 (3%)	0
***PD-L1 (%)***	*NA 10 (10%)*	*NA 3 (16%)*
0	28 (30%)	4 (25%)
1 - 49	34 (37%)	6 (38%)
50 - 100	31 (33%)	6 (38%)
***Deceased***	35 (35%)	9 (47%)
Death related to NSCLC	25 (25%)	7 (37%)
Death unrelated to NSCLC	10 (10%)	2 (11%)

## Ethics Statement

Patients with unresectable non-small cell lung cancer who did not have progressive tumour disease after definitive CRT could be included in the durvalumab EAP. The EAP was approved by the federal authority (Paul-Ehrlich-Institut, HFP Nr. 23, 22.11.2017). With written informed consent to participation in the EAP, patients agreed to the analysis of their data.

## CRediT Author Statement

**Martin Faehling:** Conceptualization, methodology, formal analysis, data collection, data curation, writing - original draft & editing, data presentation project administration. **Christian Schumann:** Data collection, writing - review & editing. **Petros Christopoulos:** Data collection, writing - review & editing. **Petra Hoffknecht:** Data collection, writing - review & editing. **Jürgen Alt:** Data collection, writing - review & editing. **Marlitt Horn:** Data collection, writing - review & editing. **Stephan Eisenmann:** Data collection, writing - review & editing. **Anke Schlenska-Lange:** Data collection, writing - review & editing. **Philipp Schütt:** Data collection, writing - review & editing. **Felix Steger:** Data collection, writing - review & editing. **Wolfgang M. Brückl:** Data collection, writing - review & editing. **Daniel C. Christoph:** Conceptualization, methodology, formal analysis, data collection, data curation, writing - original draft & editing, data presentation project administration

## Declaration of Competing Interest

Martin Faehling received speaker's honoraria and participated as PI in clinical trials of AstraZeneca, Roche, MSD, and BMS.

Christian Schumann received speaker's honoraria and participated in clinical trials by AstraZeneca, BMS, Boehringer, MSD, Pfizer, Roche, Takeda.

Petros Christopoulos received research funding from AstraZeneca, Novartis, Roche, Takeda, and advisory board/lecture fees from AstraZeneca, Boehringer Ingelheim, Chugai, Novartis, Pfizer, Roche, Takeda.

Petra Hoffknecht does not report any COIs.

Jürgen Alt received speaker's honoraria by AstraZeneca.

Marlitt Horn does not report any COIs.

Stephan Eisenmann received speaker's honoraria by AstraZeneca.

Anke Schlenska-Lange does not report any COIs.

Philipp Schütt does not report any COIs.

Felix Steger does not report any COIs.

Wolfgang M. Brückl received honoraria for consulting from AstraZeneca, BMS, Boehringer Ingelheim, Celgene, Lilly, MSD, Pfizer and Roche Pharma.

Daniel C. Christoph received speaker's honoraria and participated as PI in clinical trials of AstraZeneca, Roche, MSD, Boehringer, and BMS.

The results of our study were not influenced by the reported competing interests.

## Appendix Supplementary materials

Image, application 1

Image, application 2

Image, application 3
