# Supplementary material for: Durvalumab after definitive chemoradiotherapy in locally advanced NSCLC: Data of the German EAP
Source: Data Brief. 2020 Dec 5;34:106556. doi: 10.1016/j.dib.2020.106556 (PMC7750486; doi:10.1016/j.dib.2020.106556)
Supplement: Supplementary file 1 [file mmc1.docx]

# **Last patient contact before start of durvalumab treatment**

Date of last contact (dd.mmm.yyyy)

## **Patient`s demography**

Year of birth (XXXX)

Gender male / female

Height (cm) _____
Weight (kg) _____

## **ECOG performance status before start of durvalumab treatment**

ECOG □ not determined

0: Fully active, able to carry on all pre-disease performance without restriction □

1: Restricted in physically strenuous activity but ambulatory and able to carry out work

of a light or sedentary nature, e.g., light house work, office work □

2: Ambulatory and capable of all selfcare but unable to carry out any work activities; up

and about more than 50% of waking hours □

3: Capable of only limited selfcare; confined to bed or chair more than 50% of waking hours □

4: Completely disabled; cannot carry on any selfcare; totally confined to bed or chair □

5: Dead

## **Smoking history**

Did the patient smoke or does the patient still smoke? yes / no

If yes: How many pack years? ____

## **History of the primary tumor**

Date of first diagnosis (dd.mmm.yyyy) of NSCLC

TNM staging at time of first diagnosis

T: ____

N: ____

M: ____

## **Biomarker before start of durvalumab treatment**

PD-L1 testing performed? Yes/No/unknown

If yes:

Results?: negative/positive/unknown cut-off (percentage)?: ____________

Ratio of PD-L1 positive cells determined? Yes/No

Percentage of PD-L1 positive tumor cells: ____________

Percentage of PD-L1 positive immune cells: ____________

PD-L1 antibody clone used: ________________________

## **Previous radiotherapy used before start of durvalumab treatment**

Which kind of radiotherapy did the patient receive before start of durvalumab treatment: simultanously/sequentially

How high was the administered radiation dose:_________________________ Gy

## **Previous systemic tumor therapy before start of durvalumab treatment**

Which kind of systemic treatment did the patient receive before start tof durvaumab treatment?

1. Simultanously performed chemoradiotherapy including_______________________________
2. Induction chemotherapy including_________________________________________ and

definitive chemoradiotherapy including ___________________________________________

| Line | Regimen | Best response | Best response according to | Start of treatment | End of treatment |
| --- | --- | --- | --- | --- | --- |
| 1 | ______________ | CR / PR / SD / PD | WHO / RECIST / clinical / irRC |  |  |
| 2 | ______________ | CR / PR / SD / PD | WHO / RECIST / clinical / irRC |  |  |

## **Start of durvalumab treatment**

Date of first durvalumab infusion (dd.mmm.yyyy)

# **Clinical / radiographic control after start of durvalumab treatment**

Date of last patient contact (dd.mmm.yyyy)

## **Patient`s demography after completion of durvalumab treatment**

Weight (kg) _____

## **ECOG performance status after completion o durvalumab treatment**

ECOG □ not determined

0: Fully active, able to carry on all pre-disease performance without restriction □

1: Restricted in physically strenuous activity but ambulatory and able to carry out work

of a light or sedentary nature, e.g., light house work, office work □

2: Ambulatory and capable of all selfcare but unable to carry out any work activities; up

and about more than 50% of waking hours □

3: Capable of only limited selfcare; confined to bed or chair more than 50% of waking hours □

4: Completely disabled; cannot carry on any selfcare; totally confined to bed or chair □

5: Dead

## **Tumor assessment after end of durvalumab treatment**

After start of durvalumab treatment, did new metastasis occur? Yes / No

If yes, please specify location.

Liver □

Brain □

Bone □

Contralateral lung □

Adrenal gland / Kidney □

Lymph node metastasis outside the thorax □

Soft tissue or skin metastasis □

Malignant pleural effusion / pericardial effusion □

## **Tumor assessment after completion of durvalumab treatment**

**After start of durvalumab treatment, was a clinical tumor assessment performed?** Yes / No

If yes:

Date of last patient contact (dd.mmm.yyyy)

Clinical tumor assessment Complete Remission / Partial Remission /
 Disease Stabilization /
 Mixed Response /
 Progressive Disease

**After start of durvalumab treatment was tumor assessment using RECIST performed?**  yes/no

If yes:

Date of the most recent tumor assessment using RECIST (dd.mmm.yyyy)

Response according to RECIST Complete response (CR) /
 Partial response (PR) /
 Stable Disease (SD) /
 Progressive Disease (PD)

**After start of durvalumab treatment, was immune-related tumor assessment using irRC performed?** Yes / no

If yes:

Date of the most current immune-related tumor assessment using irRC (dd.mmm.yyyy)

Tumor burden (irRC) Immune-related Complete Response (irCR) /
 Immune-related Partial Response (irPR) /
 Immune-related Stable Disease (irSD) /
 Immune-related Progressive Disease (irPD)

## **Durvalumab-Therapie**

Was durvalumab treatment completed? Yes / No

If yes:

Reason for completion

□ regular completion after one year or treatment

□ death

Please specify date of death (dd.mmm.yyyy)

□ Progression

Please specify date of progression (dd.mmm.yyyy)

□ Adverse event caused by durvalumab

□ Adverse event not caused by durvalumab

□ Patient`s decision

□ Lost-to-follow up

# **Adverse events after start of durvalumab treatment**

Adverse event (term) ____________________________

Start (dd.mmm.yyyy):

End (dd.mmm.yyyy):

Intensity / CTC Grade □ low / Grad I
□ moderate / Grad II
□ serious / Grad III
□ very serious / Grad IV
CTC-Grad: _____

Serious adverse events (SAEs) are every life-threatening or deadly events resulting in inpatient care or prolongation of preexisting outpatient therapy, persisting or serious disability or invalidity, congenital anomaly or birth defect oder medically relevant.

Is the AE serious? □ yes □ no

Reason for category „serious“: □ deadly
□ life-threatening
□ inpatient care or prolngation
□ persisting or relevant handicap invalidity
□ medically relevant event*
Please specifiy reason for assessment of medically relevant:
_________________________________________________________________

*: i.e. an event, which is not immediately deadly or life-threatening and which does not lead to inpatient care, but – based on adaequately performed medically or scientific assessment, disstresses the patient -. Medically relevant is any AE leading to intervention/treatment, which results in any medically or surgically intervention or therapy to avoid development of a serious adverse event (SAE)

Description of the event:

Outcome: □ completely recovered
□ improved
□ improved with persisting damage
□ not recoverd
□ dead (Please specifiy reason for death)

□ unknown

Association with durvalumab: □ suspected cause
□ no relationship
□ at present not evaluable

Reason for AE: □ immune-modulated event

□ infections event

□ tumor-mediated event

□ other reason

□ unknown

Therapy focussing on durvalumab: □ no measures
□ postponement of medication
□ continuous abrogation of medication
□ unknown

Administration of corticosteroids or immune-suppressive agents: Yes / No / Unknown

**Event-relevant additonal findings from clinical or laboratory examinations:**

No relevant results □

unknown □

If durvalumab was stopped, was further durvalumab treatment started again □ Yes □ No

Did the adverse event occur again? □ Yes □ No
