# Supplementary material for: Durvalumab after definitive chemoradiotherapy in locally advanced NSCLC: Data of the German EAP
Source: Data Brief. 2020 Dec 5;34:106556. doi: 10.1016/j.dib.2020.106556 (PMC7750486; doi:10.1016/j.dib.2020.106556)
Supplement: Supplementary file 2 [file mmc2.pdf]

Last patient contact before start of durvalumab treatment

Date of last contact (dd.mmm.yyyy)

Patient`s demography

Year of birth (XXXX)

Gender male / female

Height (cm) _____

Weight (kg) _____

ECOG performance status before start of durvalumab treatment

ECOG ☐ not determined

0: Fully active, able to carry on all pre-disease performance without restriction ☐

1: Restricted in physically strenuous activity but ambulatory and able to carry out work of a light or sedentary nature, e.g., light house work, office work ☐

2: Ambulatory and capable of all selfcare but unable to carry out any work activities; up and about more than 50% of waking hours ☐

3: Capable of only limited selfcare; confined to bed or chair more than 50% of waking hours ☐

4: Completely disabled; cannot carry on any selfcare; totally confined to bed or chair ☐

5: Dead

Smoking history

Did the patient smoke or does the patient still smoke? yes / no

If yes: How many pack years? ____

History of the primary tumor

Date of first diagnosis (dd.mmm.yyyy) of NSCLC

TNM staging at time of first diagnosis

T: ____
N: ____
M: ____

Biomarker before start of durvalumab treatment

PD-L1 testing performed? Yes/No/unknown

If yes:

Results?: negative/positive/unknown cut-off (percentage)?: ____________
Ratio of PD-L1 positive cells determined? Yes/No
  Percentage of PD-L1 positive tumor cells: ____________
  Percentage of PD-L1 positive immune cells: ____________
  PD-L1 antibody clone used: ________________________

Previous radiotherapy used before start of durvalumab treatment

Which kind of radiotherapy did the patient receive before start of durvalumab treatment: simultanously/sequentially

How high was the administered radiation dose:_________________________ Gy

Previous systemic tumor therapy before start of durvalumab treatment

Which kind of systemic treatment did the patient receive before start tof durvaumab treatment?

1) Simultanously performed chemoradiotherapy including_______________________________
2) Induction chemotherapy including____ and definitive chemoradiotherapy including ____

Line	Regimen	Best response	Best response according to	Start of treatment	End of treatment
1	______________	CR / PR / SD / PD	WHO / RECIST / clinical / irRC		
2	______________	CR / PR / SD / PD	WHO / RECIST / clinical / irRC		

Start of durvalumab treatment

Date of first durvalumab infusion (dd.mmm.yyyy)

Clinical / radiographic control after start of durvalumab treatment

Date of last patient contact (dd.mmm.yyyy)

Patient`s demography after completion of durvalumab treatment

Weight (kg) _____

ECOG performance status after completion o durvalumab treatment

ECOG ☐ not determined

0: Fully active, able to carry on all pre-disease performance without restriction ☐
1: Restricted in physically strenuous activity but ambulatory and able to carry out work of a light or sedentary nature, e.g., light house work, office work ☐
2: Ambulatory and capable of all selfcare but unable to carry out any work activities; up and about more than 50% of waking hours ☐
3: Capable of only limited selfcare; confined to bed or chair more than 50% of waking hours ☐
4: Completely disabled; cannot carry on any selfcare; totally confined to bed or chair ☐
5: Dead

Tumor assessment after end of durvalumab treatment

After start of durvalumab treatment, did new metastasis occur? Yes / No

If yes, please specify location.

Liver ☐
Brain ☐
Bone ☐
Contralateral lung ☐
Adrenal gland / Kidney ☐
Lymph node metastasis outside the thorax ☐
Soft tissue or skin metastasis ☐
Malignant pleural effusion / pericardial effusion ☐

Tumor assessment after completion of durvalumab treatment

After start of durvalumab treatment, was a clinical tumor assessment performed? Yes / No

If yes:

Date of last patient contact (dd.mmm.yyyy)

Clinical tumor assessment — Complete Remission / Partial Remission / Disease Stabilization / Mixed Response / Progressive Disease

After start of durvalumab treatment was tumor assessment using RECIST performed? yes/no

If yes:

Date of the most recent tumor assessment using RECIST (dd.mmm.yyyy)

Response according to RECIST — Complete response (CR) / Partial response (PR) / Stable Disease (SD) / Progressive Disease (PD)

After start of durvalumab treatment, was immune-related tumor assessment using irRC performed? Yes / no

If yes:

Date of the most current immune-related tumor assessment using irRC (dd.mmm.yyyy)

Tumor burden (irRC) — Immune-related Complete Response (irCR) / Immune-related Partial Response (irPR) / Immune-related Stable Disease (irSD) / Immune-related Progressive Disease (irPD)

Durvalumab-Therapie

Was durvalumab treatment completed? Yes / No

If yes:

Reason for completion

☐ regular completion after one year or treatment

☐ death

Please specify date of death (dd.mmm.yyyy)

☐ Progression

Please specify date of progression (dd.mmm.yyyy)

□ Adverse event caused by durvalumab

□ Adverse event not caused by durvalumab

☐ Patient`s decision

☐ Lost-to-follow up

Adverse events after start of durvalumab treatment

Adverse event (term) ______________________________

Start (dd.mmm.yyyy):

End (dd.mmm.yyyy):

Intensity / CTC Grade
☐ low / Grad I
☐ moderate / Grad II
☐ serious / Grad III
☐ very serious / Grad IV
CTC-Grad: _____

Serious adverse events (SAEs) are every life-threatening or deadly events resulting in inpatient care or prolongation of preexisting outpatient therapy, persisting or serious disability or invalidity, congenital anomaly or birth defect oder medically relevant.

Is the AE serious? ☐ yes ☐ no

Reason for category „serious“:
☐ deadly
☐ life-threatening
☐ inpatient care or prolngation
☐ persisting or relevant handicap invalidity
☐ medically relevant event*
Please specifiy reason for assessment of medically relevant:
____
____

*: i.e. an event, which is not immediately deadly or life-threatening and which does not lead to inpatient care, but – based on adaequately performed medically or scientific assessment, disstresses the patient -. Medically relevant is any AE leading to intervention/treatment, which results in any medically or surgically intervention or therapy to avoid development of a serious adverse event (SAE)

Description of the event:

Outcome:

☐ completely recovered
☐ improved
☐ improved with persisting damage
☐ not recoverd
☐ dead (Please specifiy reason for death)
☐ unknown

Association with durvalumab:

☐ suspected cause
☐ no relationship
☐ at present not evaluable

Reason for AE:

☐ immune-modulated event
☐ infections event
☐ tumor-mediated event
☐ other reason
☐ unknown

Therapy focussing on durvalumab:

☐ no measures
☐ postponement of medication
☐ continuous abrogation of medication
☐ unknown

Administration of corticosteroids or immune-suppressive agents: Yes / No / Unknown

Event-relevant additonal findings from clinical or laboratory examinations:

No relevant results ☐
unknown ☐

If durvalumab was stopped, was further durvalumab treatment started again

Did the adverse event occur again? ☐ Yes ☐ No
